# Minimal uranium accumulation in lymphoid tissues following an oral 60-day uranyl acetate exposure in male and female C57BL/6J mice

**DOI:** 10.1371/journal.pone.0205211

**Published:** 2018-10-24

**Authors:** Alicia M. Bolt, Sebastian Medina, Fredine T. Lauer, Huan Xu, Abdul-Mehdi Ali, Ke Jian Liu, Scott W. Burchiel

**Affiliations:** 1 College of Pharmacy, Department of Pharmaceutical Sciences, The University of New Mexico, Albuquerque, New Mexico, United States of America; 2 School of Pharmacy, Department of Pharmaceutical Sciences, East China University of Science and Technology, Shanghai, China; 3 Department of Earth and Planetary Science, The University of New Mexico, Albuquerque, New Mexico, United States of America; University of Texas Medical Branch at Galveston, UNITED STATES

## Abstract

High levels of uranium (U) exist in soil, water, and air in the Southwestern United States due, in part, to waste generated from more than 160,000 abandoned hard rock mines located in this region. As a result, many people living in this region are chronically exposed to U at levels that have been linked to detrimental health outcomes. In an effort to establish a relevant *in vivo* mouse model for future U immunotoxicity studies, we evaluated the tissue distribution of U in immune organs; blood, bone marrow, spleen, and thymus, as well as femur bones, kidneys, and liver, following a 60-d drinking water exposure to uranyl acetate (UA) in male and female C57BL/6J mice. Following the 60-d exposure, there was low overall tissue retention of U (<0.01%) at both the 5 and the 50 ppm (mg/L) oral concentrations. In both male and female mice, there was limited U accumulation in immune organs. U only accumulated at low concentrations in the blood and bone marrow of male mice (0.6 and 16.8 ng/g, respectively). Consistent with previous reports, the predominant sites of U accumulation were the femur bones (350.1 and 399.0 ng/g, respectively) and kidneys (134.0 and 361.3 ng/g, respectively) of male and female mice. Findings from this study provide critical insights into the distribution and retention of U in lymphoid tissues following chronic drinking water exposure to U. This information will serve as a foundation for immunotoxicological assessments of U, alone and in combination with other metals.

## Introduction

Waste from abandoned hard rock mines located in the Southwestern United States has contributed to metal contamination of soil, water, and air sources throughout the region. Unfortunately, these abandoned mines are frequently found on tribal lands. In the Navajo Nation alone, there are more than 500 abandoned U mines [1]. High levels of U and other metals have been reported in water, air, and soil sources from the Navajo Nation and could be a potential source of chronic exposure and toxicity for individuals living in this region [1–3]. The vast majority of people living in the Navajo Nation and surrounding rural areas rely on unregulated drinking water sources (i.e. well water) that often contains elevated levels of U and other metals [4]. A recent survey of unregulated water sources across the Navajo Nation, 75% of the sources had detectable levels of U and 12.5% had U concentrations exceeding the U.S. Environmental Protection Agency (U.S. EPA) and World Health Organization maximum contaminant levels for U (30 parts per billion; ppb; μg/L) [1, 5, 6].

Knowledge of the biological fate and tissue distribution of U following oral exposure are important to accurately assess toxicity. Based on data from animal studies, U is poorly absorbed regardless of exposure route and rapidly excreted from the body, resulting in low tissue retention [7–10]. Animal studies and epidemiological data indicate that bone and kidney are the primary sites of tissue accumulation and toxicity of U [11–15]. However, very little is known about the distribution and associated toxicity of U in lymphoid tissues following drinking water exposure, which is one of the primary routes of exposure in humans.

The objective of this study was to evaluate the distribution of U, in the form of UA, following a 60-d drinking water exposure in male and female C57BL/6J mice. We were particularly interested in the accumulation of U in lymphoid tissues because of our interests in U immunotoxicity. This information will be used in conjunction with immunotoxicity studies to gain an understanding of the negative health effects of U exposure on immune endpoints in human populations.

## Materials and methods

### *In vivo* mouse exposures

All animal experiments were approved by the University of New Mexico (UNM) Office of Animal Care Compliance Committee (Albuquerque, NM). Wild-type C57BL/6J mice (male and female, 6 weeks old) were purchased from Jackson Laboratory (Bar Harbor, MA) and housed in the UNM Health Science Center (HSC) Animal Resource Facility. Mice were given food and water *ad libitum*. For *in vivo* U exposures, mice were divided into 3 groups with 7 mice per group: control (tap water), 5 or 50 parts per million (ppm; mg/L). To model environmental exposures to U from abandoned U mine waste, we used the soluble uranyl acid, UA, which is U^235^ depleted. Stock solutions of UA (>95% purity, UO_2_(OCOCH_3_)_2_·2H_2_O; U^238^–99.9%, U^235^–0.1%; Electron Microscopy Sciences, Hatfield, PA) were prepared in MilliQ water and used throughout the duration of the experiment. UA was diluted into drinking water to a final concentration of either 5 or 50 ppm U and replaced once per week. The concentration of U used in our study represents elemental U as opposed to the dehydrate compound (1.8 g UO_2_(OCOCH_3_)_2_·2H_2_O = 1 g U).

The concentrations of U used in this study were systematically selected based on relevance to environmental U exposures on the Navajo Nation in the Southwestern United States as well as other regions affected by U contamination. On the Navajo Nation, U in unregulated water sources has been measured at concentrations as high as 269 ppb, approximately 9 times higher than the U.S. EPA regulated limit of 30 ppb [3, 16]. U measured in soils collected from the Navajo Nation have been reported at levels ranging from 2,200 to 6,600 mg/kg (ppm) [3], approximately 700 to 2,000 times greater than the average U content in non-contaminated United States soils (~3 mg/kg; ppm), respectively [7]. U contamination of drinking and ground water sources in other regions of the world have been reported as high as 700 ppb and 20 ppm, respectively [5, 17]

After a 1-week acclimation period, mice were exposed to tap water or UA in tap water for 60-days. We observed no changes in animal weight or physical appearance in the UA-exposed group. After the 60-d exposure, mice were euthanized by CO_2_ asphyxiation followed by cardiac puncture. Tissues (whole blood, liver, spleen, thymus, femurs, and kidney) were harvested, weighed, and stored frozen at -80˚C prior to acid digestion and U analysis. For femur bone samples, bone marrow cells were flushed from femur bones; empty bones were weighed and frozen at -80˚C until acid digestion and U analysis. Single cells suspensions of bone marrow, spleen, and thymus were generated and counted. Immune cells from the bone marrow, spleen, and thymus were pelleted and frozen at -80˚C until acid digestion for U concentration analysis.

### Uranium tissue analysis

Uranium concentrations in mouse tissues (femur bones, kidneys, liver, whole blood, bone marrow, spleen, and thymus) were quantified using PerkinElmer NexION 300D Inductively Coupled Plasma Mass Spectrometry (ICP/MS). Whole tissues, or isolated cells were digested with 70% trace metal grade nitric acid and heated up to 95 ˚C, when necessary to completely digest tissue. Digested samples were diluted to a final volume of 10 mL with ultra-high purity, ~2% nitric acid. The diluted samples were 0.45 micron filtered before analysis. The ICP/MS was optimized to include the use of anhydrous ammonia in the Dynamic Reaction Cell (DRC), which significantly minimizes mass interferences. Calibrated, and quality control (QC) samples were analyzed to validate the calibration curve before analyzing the samples. During sample analysis, continuing quality control samples were analyzed after each 20 sample batch to verify calibration of the instrument. Data were exported and reported as mean U concentration (ng) per g of tissue weight. For the bone marrow samples, total number of bone marrow cells isolated was converted into g tissue weight from a five-point standard curve generated based on the weights of pelleted bone marrow cells (5–60 x 10^6^ cells). For the immune cell analyses (bone marrow, spleen, and thymus), data were also reported as mean U concentration (pg) per 10^6^ million cells (S1 Fig). Whole blood data were reported as ng U/g tissue weight for consistency and to eliminate pipetting error from the analysis. The Method Detection Limit (MDL) of U for the ICP/MS analysis was 0.010 ppb.

### Statistics

Data were analyzed using Sigma Plot statistical software version 12.5 (San Jose, CA) using One-Way ANOVA followed by a Dunnett’s t-test comparing each treatment group to the control for each tissue or cell sample analyzed. When the data were non-normally distributed, a Kruskal-Wallis ANOVA was used. For two group comparisons, data were analyzed using a Student’s t-test. Statistically significant outliers (Grubb’s Test; p < 0.05) were excluded from ICP/MS data. Regardless of the statistical approach used, all differences between control and treatment groups were considered statistically significant at *p*<0.05.

## Results

Due to our interest in U immunotoxicity, the objective of this study was to evaluate the concentrations of U found in immune tissues following an oral U exposure. Of particular interest were whole blood, bone marrow, spleen, and thymus. We exposed male and female C57BL/6J mice to 5 and 50 ppm U, in the form of UA (U^235^ depleted U), in drinking water for 60-days. At the highest dose of U, male and female mice had a total intake of 9,468 and 9,282 μg of U over the course of the 60-d study, respectively (Table 1). There were no differences in mouse body weights, tissue weights, or total cell recoveries for any of the tissues analyzed (data not shown). Concentrations of U in tissues were measured in tissues and cells by ICP/MS following the 60-d oral exposure.

**Table 1:** Water concentration (based on ICP/MS results), water consumption, daily and total uranium intake, total uranium accumulated in tissues (based on ICP/MS results)*. n = 7/ group. mean values ± S.D. of the mean.

	Dose Group	Water U Conc. (ppm)	Water Intake / Day / Mouse (mL)	Daily U Intake / Mouse (μg)	Total U Intake / Mouse (μg)	Total U Accumulated in Tissues (ng)
**Male**	**Control**	0.003 ± 0.01	3.34 ± 0.61	0.01	0.60	7.78 ± 10.4
	**5 ppm**	5.19 ± 0.28	2.75 ± 0.21	14.34	860.40	23.78 ± 18.8
	**50 ppm**	53.13 ± 2.15	2.97 ± 0.16	157.80	9468.00	100.25 ± 13.2
**Female**	**Control**	0.004 ± 0.0001	2.94 ± 0.09	0.01	0.60	1.54 ± 0.2
	**5 ppm**	5.21 ± 0.10	2.61 ± 0.14	13.60	816.00	11.83 ± 4.0
	**50 ppm**	51.74 ± 2.11	2.99 ± 0.12	154.70	9282.00	137.42 ± 39.4

* Total U Accumulated in Tissues (ng) represents the cumulative total of all U measured by ICP/MS for the 7 tissues analyzed.

**Fig 1:**
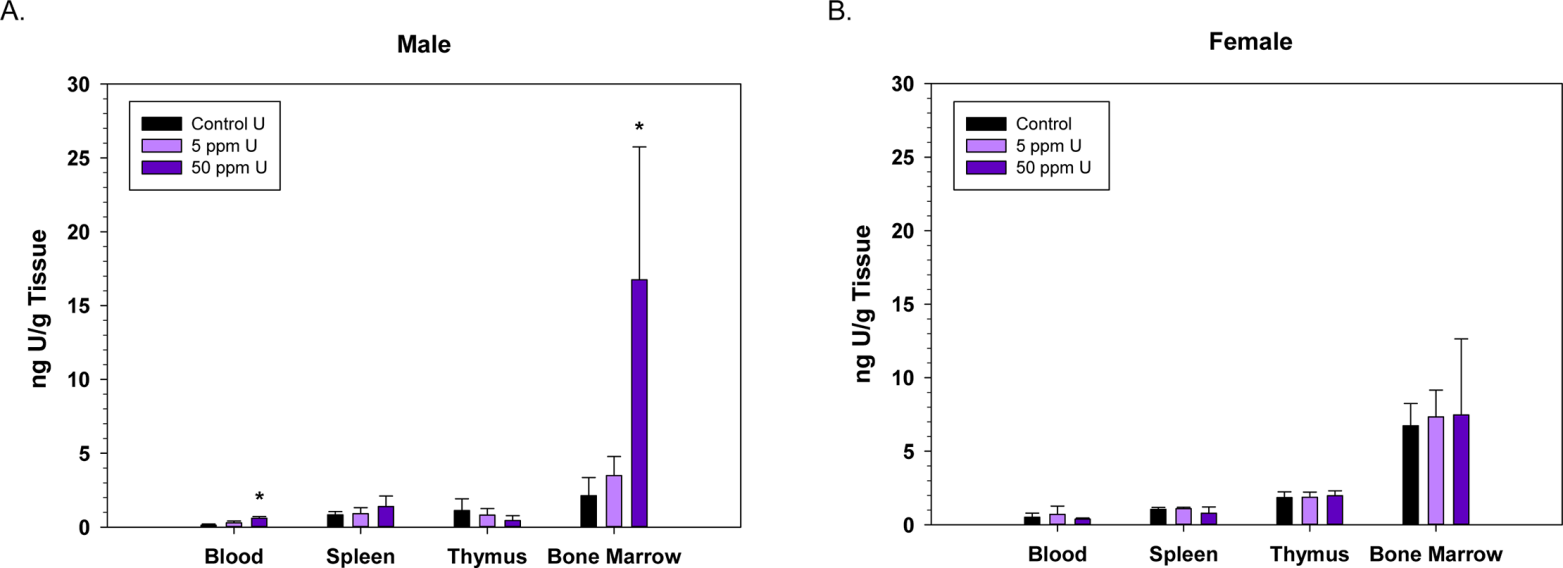
Uranium is detected in the blood and bone marrow of male mice following a 60-d oral exposure. Uranium concentrations in single cell suspensions (bone marrow, spleen, and thymus) and whole blood were analyzed by ICP/MS, following an oral 60-d exposure to uranyl acetate. Graph indicates mean g uranium/g of tissue +/- S.D. of mean for each cell type in male (A) and female (B) mice. n = 6 or 7 group. *p< 0.*05*; One-Way ANOVA or Kruskal Wallis ANOVA, as appropriate.

**Fig 2:**
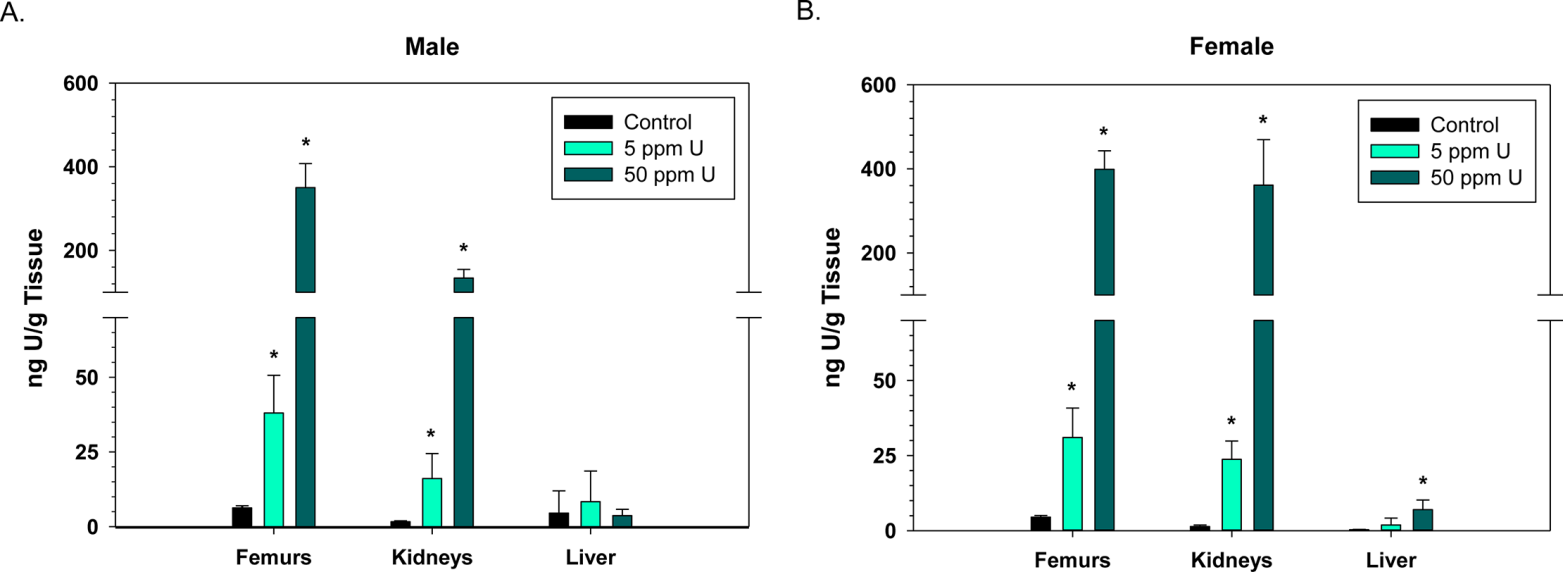
Uranium accumulates in the bone and kidney following a 60-d oral exposure. Uranium concentrations in harvested mouse tissues (femur bones, kidneys, and liver) were analyzed by ICP/MS, following an oral 60-d exposure to uranyl acetate. Graph indicates mean ng uranium/g of tissue +/- S.D. of mean for each tissue in male (A) and female (B) mice. n = 6 or 7/ group. *p< 0.*05*; One-Way ANOVA or Kruskal Wallis ANOVA, as appropriate.

In both male and female mice, there was no significant accumulation of U in the spleen or thymus (Fig 1A & 1B). Males had low, but significantly elevated levels of U in whole blood (0.6 ng/g) and bone marrow (16.8 ng/g) at the 50 ppm exposure dose (Fig 1A). In addition, for the immune cell analyses (bone marrow, spleen, and thymus) the data are also reported as mean U concentration (pg) per 10^6^ million cells (S1 Fig). Uranium predominantly accumulated in the femur bones and kidneys (Fig 2A & 2B) at both the 5 and 50 ppm exposures. In 50 ppm exposed male mice, 350.1 ng/g U was measured in the bone and 134.0 ng/g U in the kidneys (Fig 2A). In 50 ppm exposed female mice, 399.0 ng/g U was measured in the bone and 361.3 ng/g in the kidneys (Fig 2B). Female mice also had low, but significantly increased levels of U in the liver (7.0 ng/g) (Fig 2B).

**Fig 3:**
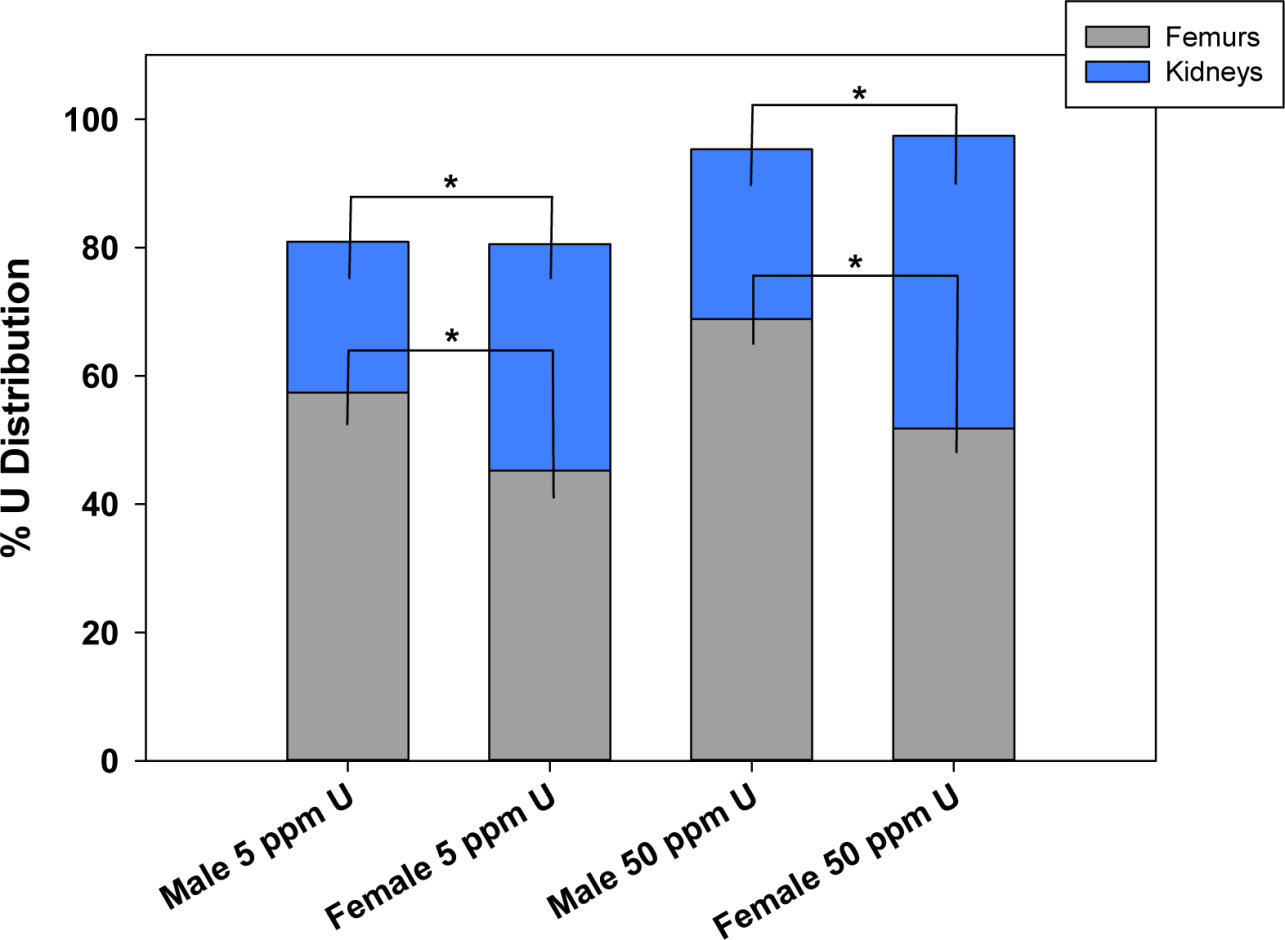
The percentage of total uranium distributed in femur bones and kidneys in male and female mice. The percentage of total uranium located in the femur bones and kidneys was calculated from ICP/MS data. Graph indicates mean percent uranium distribution +/- S.D. of the mean for each tissue in male and female mice. n = 6 or 7/ group. *p< 0.*05*; Students t-test, comparing male and female mice at the 5 and 50 ppm uranium concentrations.

The overall percentage of U accumulation was evaluated in the two primary sites of U deposition (bone and kidney), which accounted for between 80–95% of the total U distributed in the tissues measured. At both the 5 and 50 ppm exposure levels, females displayed a significantly higher overall percentage of U accumulation in the kidneys; whereas, males had a significantly higher percentage of U accumulation in femur bones (Fig 3). Collectively, these results show that males and females exhibit similar patterns of U tissue distribution, with very little U accumulation in lymphoid tissues.

## Discussion

In an effort to gain understanding of the distribution of U in immunologically relevant tissues, male and female C57BL/6J mice were exposed to 5 and 50 ppm U for 60-days in their drinking water. Following the 60-d exposure, resulting levels of U were measured by ICP/MS in several major immune organs (i.e. whole blood, spleen, thymus, bone marrow) as well as other known sites of known U deposition.

Based on this 60-d U distribution analysis, very little U was found to accumulate in the immune organs of male and female mice. To our knowledge, this is the first animal study to investigate the distribution of U in immune organs of males and females following drinking water exposure to UA. One previous study evaluated the accumulation of U in immune organs of mice, but they only included the spleen and thymus and did not consider females [18]. Contrary to our findings, Hao et al., (2013), reported that male mice exposed to uranyl nitrate in feed for 120-d had significant U accumulation in the spleen and thymus. At doses similar to those used in our study (3 and 30 mg/kg; ppm), the investigators reported a 5–6 fold increase (relative to unexposed mice) in U concentrations measured in the spleen and thymus. However, differences in exposure duration (120-d vs. 60-d), the chemical species of U utilized (uranyl nitrate vs. UA), and strain of mice (Kunming vs. C57BL/6J mice) could have been factors that contributed to the discrepancy between our results.

Overall tissue retention of U was low in both male and female mice in this study (<0.01%; Table 1). This finding is consistent with previous U pharmacokinetic studies in rodents and humans that document very little gastrointestinal absorption and rapid excretion of U [19]. In addition, similar to previous studies, we also found that the predominate sites of U accumulation in male and female mice were the bone and kidneys [11, 12, 18, 20–23].

Several factors have been identified to significantly influence the tissue retention of U. It has been reported that fasting animals absorb 10 times more U than non-fasting animals [24]. It has also been found that dietary iron (supplement and deficiency) can significantly increase U absorption [10, 25]. This information is particularly important with regard to human U exposure and suggest that, although overall U absorption is low, under certain dietary conditions such as fasting or changes in iron intake can affect U retention and potential toxicity. The influence of changes in dietary conditions, such as iron and their impact on the retention of U in biological tissues will be a topic of our future investigations. In addition, another important factor to consider with regards to tissue retention of U is that in biological fluids, the uranyl ion is complexed with anions such as citrate and bicarbonate [26, 27]. Understanding how these complexed forms of U exist at different biological pHs and how this affects U tissue absorption is also necessary to better understand the fate of U once it enters the body.

Since another major route of human exposure to U is inhalation, it will also be important to perform future studies to determine whether oral vs. inhalation exposure routes produce differential effects on U immune tissue distribution and immunotoxicity.

The tissue distribution of U in male and female mice was examined to identify potential sex differences in U accumulation. Importantly we found that males had low, but notable accumulation of U in immune organs whereas females did not. In addition, while the two primary sites of U accumulation did not differ between males and females (bone and kidney); there were differences between males and females in the proportion of U that accumulated at those sites. Females displayed a greater proportion of U accumulation in the kidneys, whereas males had a larger percentage in the bone. These differences in accumulation may increase susceptibility to U toxicity in a sex and tissue specific manner and should be more thoroughly evaluated in future studies.

Our study presents novel findings regarding the distribution of U in lymphoid tissues of male and female mice and is of the first studies to describe the general distribution characteristics of U in males and females following environmentally relevant drinking water exposures to UA. Understanding the differential U accumulation characteristics in males and females will be used in conjunction with immunotoxicity studies to evaluate the impact of U exposure on immune system health in human populations.

## Supporting information

S1 Fig Uranium minimally accumulates in immune organs following a 60-day oral exposure. Uranium concentration in single cell suspensions (bone marrow, spleen, and thymus) were analyzed by ICP/MS, following an oral 60 d exposure to uranyl acetate. Graph indicates mean pg uranium/10^6^ cells +/- S.D. of mean for each cell type in male (A) and female (B) mice. N = 6 or 7 group. *p< 0.*05*; One-Way ANOVA or Kruskal Wallis ANOVA, as appropriate.
(TIF)
