# Supplementary figures and images for: Minimal uranium accumulation in lymphoid tissues following an oral 60-day uranyl acetate exposure in male and female C57BL/6J mice
Source: PLoS One. 2018 Oct 24;13(10):e0205211. doi: 10.1371/journal.pone.0205211 (PMC6200214; doi:10.1371/journal.pone.0205211)

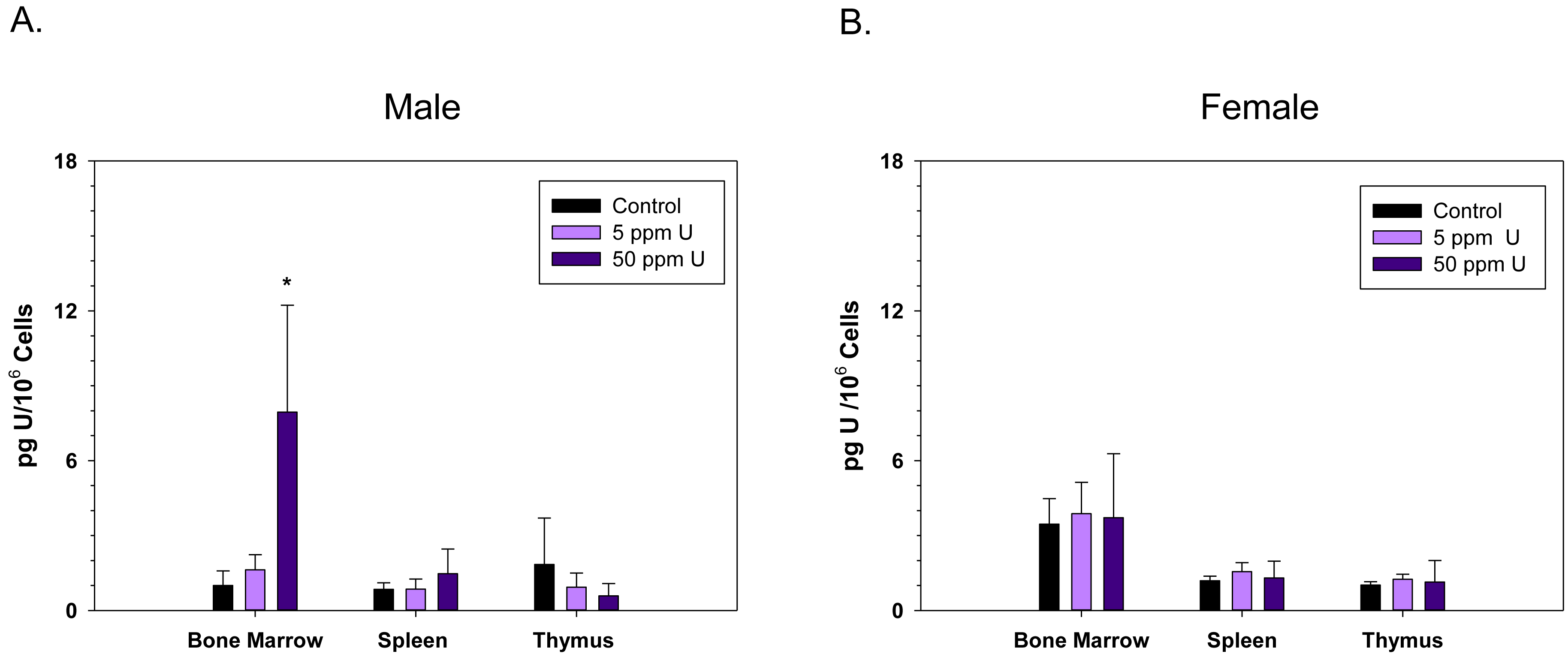

Supplement: S1 Fig — Uranium concentration in single cell suspensions (bone marrow, spleen, and thymus) were analyzed by ICP/MS, following an oral 60 d exposure to uranyl acetate. Graph indicates mean pg uranium/106 cells +/- S.D. of mean for each cell type in male (A) and female (B) mice. N = 6 or 7 group. *p< 0.05; One-Way ANOVA or Kruskal Wallis ANOVA, as appropriate. (TIF) [file pone.0205211.s001.tif]
